# Correlation between macrophage migration inhibitory factor and autophagy in *Helicobacter pylori*-associated gastric carcinogenesis

**DOI:** 10.1371/journal.pone.0211736

**Published:** 2019-02-11

**Authors:** Kichul Yoon, Nayoung Kim, Youngmi Park, Bo Kyung Kim, Ji Hyun Park, Cheol Min Shin, Dong Ho Lee, Young-Joon Surh

**Affiliations:** 1 Department of Internal Medicine, Seoul National University Bundang Hospital, Seongnam, South Korea; 2 Department of Internal Medicine and Liver Research Institute, Seoul National University College of Medicine, Seoul, South Korea; 3 Medical Research Collaborating Center, Seoul National University Bundang Hospital, Seongnam, South Korea; 4 Tumor Microenvironment Global Core Research Center, College of Pharmacy, Seoul National University, Seoul, South Korea; National Cancer Center, JAPAN

## Abstract

The role of macrophage migration inhibitory factor (MIF) and autophagy in gastric cancer is not clear. We determined *H*. *pylori* infection status of the subjects and investigated the expression of MIF and autophagy markers (Atg5, LC3A and LC3B) in human gastric tissue at baseline. Then *H*. *pylori* eradication was done for *H*. *pylori* positive patients and MIF and Atg5 levels were investigated on each follow-up for both *H*. *pylori*-eradicated and *H*. *pylori* negative patients. Baseline tissue mRNA expression of MIF, Atg5, LC3A and LC3B was measured by real-time PCR in 453 patients (control 165, gastric dysplasia 82, and gastric cancer 206). Three hundred three patients (66.9%) had *H*. *pylori* infection at the time of enrollment. Only within *H*. *pylori*-positive group, MIF level was significantly elevated in patients with cancer than in control or dysplasia groups (*P<*0.05). LC3A and LC3B levels also showed significant differences within *H*. *pylori*-positive subgroups. *H*. *pylori*-positive dysplasia subgroup showed significantly lower (LC3A) (*P*<0.05) and higher (LC3B) mRNA levels (*P*<0.05) than in other subgroups. On follow-up, within *H*. *pylori*-eradicated group, Atg5 expression increased sequentially from control to dysplasia and cancer subgroups. Multiple linear regression showed autophagy markers (LC3A, LC3B, and Atg5) directly predicted MIF level (adjusted R^2^ = 0.492, *P*<0.001). Serial follow-up showed longitudinal increase in Atg5 level in general, with constantly higher levels in *H*. *pylori*-eradicated group than in -negative group. Intestinal metaplasia (IM) group initially showed higher Atg5 expression than the IM-negative group. However, it was reversed between the groups eventually because of the lower rate of increase in IM group. These results suggest a role of MIF and autophagy markers and their interaction in *H*. *pylori*-associated gastric carcinogenesis.

## Introduction

Gastric cancer is one of the most prevalent cancer types worldwide, particularly in East Asian populations [1]. Gastric dysplasia is a direct precancerous lesion representing the penultimate stage in gastric carcinogenesis [2]. The role of *Helicobacter pylori* (*H*. *pylori*) in the development of gastric dysplasia and cancer has been extensively studied. However, the underlying mechanism in human tissue still remains elusive [3].

Macrophage migration inhibitory factor (MIF) is one of the first cytokines discovered [4]. Evidence supports the role of MIF in tumorigenesis and tumor progression, especially in the background of tumor microenvironment [5, 6]. The relationship between MIF and cancers such as non-small cell lung cancer, breast cancer, colorectal cancer, prostate cancer, esophageal cancer, hepatocellular carcinoma, and ovarian cancer has been investigated [7]. Increased epithelial and serum expression of MIF in gastric cancer suggest its diagnostic and prognostic role in gastric cancer [8, 9]. However, the role of MIF in the context of *H*. *pylori* infection, which is one of the most important causes of gastric cancer, has yet to be investigated [10]. Meta-analysis of epidemiological studies and animal models have shown that both intestinal and diffuse types of gastric cancer are equally associated with *H*. *pylori* infection [11].

Autophagy is an evolutionarily conserved catabolic process. It is morphologically characterized by the formation of double membrane autophagosomes, which control the fate of impaired organelles or unwanted cellular components for delivery to lysosomes for degradation and recycling. [12]. In gastric cancer, its role remains elusive with seemingly contradictory reports. An autophagosome marker LC3 was highly expressed in gastrointestinal cancers [13]. However, the high expression of another autophagy marker Beclin-1 was associated with favorable prognosis [14]. As autophagy plays a role in *H*. *pylori*-associated gastritis [15] it might be valuable to evaluate its role in gastric carcinogenesis. In addition, the relationship between MIF and autophagy is largely unknown except a report suggesting that cellular autophagy was induced by MIF via reactive oxygen species generation under stress [16].

We hypothesized that MIF and autophagy markers play a role in *H*. *pylori*-associated gastric carcinogenesis and probably interact with each other. The aim of this prospective study is to investigate the correlation between molecular markers and histopathology according to *H*. *pylori* status. In addition, we serially followed MIF and Atg5 levels to determine any longitudinal variation in the cytokine levels after *H*. *pylori* eradication.

## Methods

### Study population

Four hundred and fifty-three patients who underwent upper endoscopy at Seoul National University Bundang Hospital from February 2006 to February 2014 were enrolled. Biopsy and *H*. *pylori* tests were performed at baseline and also at each follow-up. Exclusion criteria were: concomitant renal or chronic hepatic disease, previous gastric surgery, current pregnancy or lactation, and treatment with steroids or nonsteroidal anti-inflammatory drugs. This study was approved by the Institutional Review Board of the Seoul National University Bundang Hospital, Korea (IRB Number: B-1409/266-302).

### *H*. *pylori* tests and histology

At each endoscopic examination, five biopsy specimens were obtained from the antrum and the mid-body of the stomach, respectively [17], performed solely by Nayoung Kim. Tissue sections were stained with hematoxylin and eosin (H&E) stain for histological examination of atrophic gastritis and intestinal metaplasia (IM) according to Updated Sydney Classification System and modified Giemsa for confirmation of the presence of *H*. *pylori*. *H*. *pylori* status was additionally assessed by rapid urease test [*Campylobacter* like organism (CLO) test, Delta West, Bentley, Australia] and culture studies. Protocols for the biopsy-based tests were described previously [18]. Specific IgG for *H*. *pylori* was screened using an enzyme-linked immunosorbent assay (ELISA) of each subject’s serum (Genedia *H*. *pylori* ELISA; Green Cross Medical Science Corp, Eumsung, South Korea). The Korean strain was used as antigen for the *H*. *pylori* antibody test. Each patient was asked about their history of *H*. *pylori* eradication and if all of these four tests and history of *H*. *pylori* eradication were negative, the subject was deemed *H*. *pylori*-negative, as described in detail previously elsewhere.[19].

### Quantitative real-time polymerase chain reaction

The PCR cycling procedure was performed as described in detail elsewhere previously.[20] The primer sequences are shown on the S4 Table (see online). Briefly, total RNA was extracted directly from non-cancerous corporal biopsy specimens with TRIzol reagent (Invitrogen, Carlsbad, CA, USA), and 1000 ng of RNA was reverse transcribed to complementary DNA with oligo (dT) and M-MLV reverse transcriptase (Invitrogen), according to the manufacturers’ instructions. Quantitative PCR was performed in 96-well reaction plates using 2 μl of complementary DNA in a 20 μl reaction mixture containing 2× SYBR Premix Ex Taq (Takara Bio, Otsu, Japan). Samples were run on a StepOne Plus real-time PCR instrument (Applied Biosystems, Foster City, CA Baseline expression levels of mRNA of the target gene were compared with the endogenous control β-actin using the 2^-ΔΔCT^ method [21]. For longitudinal analysis, mRNA expression levels were log-transformed via a log(1+2^-ΔCT^) [22].

### Follow-up measurements

The enrolled patients had undergone endoscopy every 18 months with *H*. *pylori* tests and histopathological examinations. Every patient with *H*. *pylori*-positive status underwent eradication therapy right after the enrollment. When the first eradication therapy failed, the 2^nd^ and 3^rd^ interventions were performed until the pathogen was eradicated [23]. Tissue samples were obtained from corpus to measure the expression of MIF and autophagy markers.

### Statistical analysis

The χ2 test and Fisher’s exact test were used for the analysis of categorical variables. To compare continuous variables, one-way ANOVA (analysis of variance) was performed followed by Games-Howell post-hoc test, based on the result of test for equality of variances. Longitudinal data were analyzed with linear mixed model using random intercept model. All analyses were performed using either SPSS (version 21.0, IBM, NY) or Stata 14/SE (Timberlake Consultants, UK)

## Results

### Subject characteristics

A total of 453 patients were enrolled (mean age 58.4 ± 13.2). The study population consisted of 273 males (60.3%) and 180 females (39.7%). Among them, 206 were diagnosed with cancer, 82 with dysplasia, and 165 control patients were included. Three hundred and three patients (66.9% of total) had current *H*. *pylori* infection at the time of enrollment: control (84 patients), gastric dysplasia (49 patients) or cancer (170 patients) (Table 1). One hundred and fifty patients (33.1%) were found *H*. *pylori*-negative according to aforementioned criteria. Most of the patients in the dysplasia group had low-grade dysplasia. (45 patients, 91.8%). Among *H*. *pylori*-positive patients, 137 patients (80.6% among cancer) with early gastric cancer (EGC), and 119 patients (70%) with intestinal type cancer were included. (Table 1). A higher ratio of male population was detected in the *H*. *pylori*-positive group than in *H*. *pylori*-negative group (*P* < 0.05) (Table 1).

**Table 1:** Baseline characteristics.

		*N*	Sex (male, %)	AGE	LGD (%)	EGC (%)	Intestinal type (%)
			*P = 0.004	^#^*P* < 0.05			
	control	84	43 (51.2%)	53.8 ± 11.7			
**HP positive**	dysplasia	49	36 (73.5%)	62.5 ± 7.5	45(91.8%)		
	cancer	170	118 (69.4%)	62.0 ± 10.9		137(80.6%)	119(70%)
*HP positive total*		*303 (66*.*9%)*	*197 (65*.*0%)*	*59*.*3 ± 11*.*3*			
	control	81	32 (39.5%)	54.6 ± 17.1			
**HP negative**	dysplasia	33	22 (66.7%)	61.9 ± 12.6	31(93.9%)		
	cancer	36	22 (61.1%)	57.5 ± 14.0		18(50%)	17(47.2%)
*HP negative total*		*150 (33*.*1%)*	*76 (50*.*7)*	*56*.*9± 15*.*7*			
	control	165	75(45.5%)	54.2±14.5			
**Total**	dysplasia	82	58(70.7%)	62.27±9.8	76(92.7%)		
	Cancer	206	140(68.0%)	60.9±11.8		155(75.2%)	136(66.0%)
	Total	453 (100%)	273 (60.3%)	58.4 ± 13.2			

Data shown in Mean ± Standard Deviation; HP, Helicobacter pylori. LGD, Low-grade dysplasia; The remainder of the dysplasia group had high-grade dysplasia; EGC: Early Gastric Cancer. The remainder of the cancer group had Advanced Gastric Cancer; Intestinal type: The remainder of the cancer group had diffuse type pathology

* *P*-value for chi-squared test for six groups

# *P*-value for equality of all means of six groups

### Tissue MIF level

**Fig 1:**
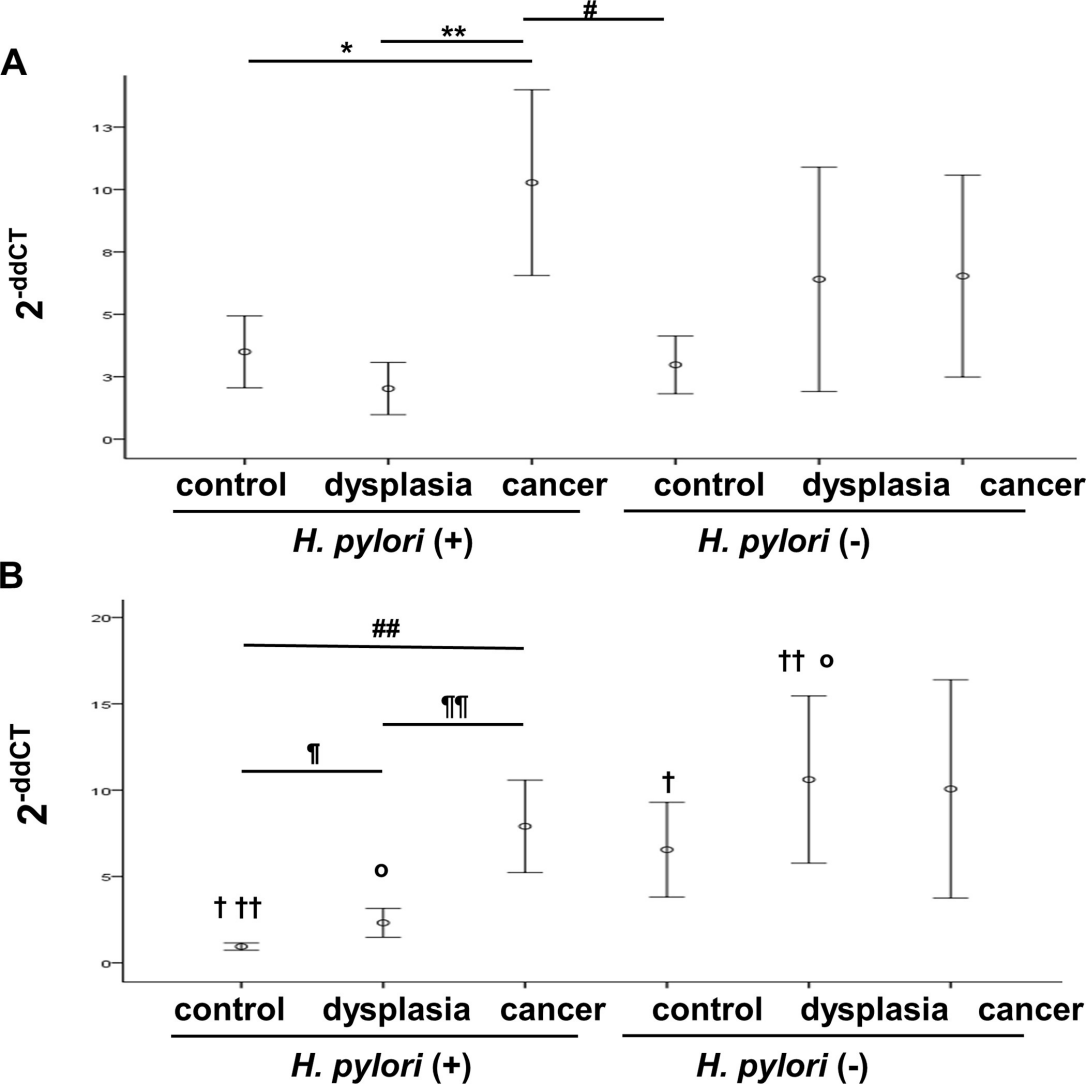
Tissue MIF and Atg5 levels. (A) In *H*. *pylori*-positive group, MIF level was significantly elevated in cancer subgroup than in control or dysplasia subgroups. (B) Within *H*. *pylori*-positive group, the Atg5 expression increased sequentially from control to dysplasia, and to cancer subgroups. All data were expressed as mean ± S.E; *P = 0.012;**,^#^*P* < 0.01, ^¶, ¶¶, ##, †, ††, o^ *P* < 0.05. The same symbols above the graph indicates the significant difference between the designated subgroups based on Games-Howell post-hoc test.

There was no significant difference in tissue MIF level according to age (Adjusted R^2^ = 0.003, *P* > 0.05) or sex (*P* > 0.05, S3 Table). When study population was divided into cancer and non-cancer groups regardless of *H*. *pylori* status, the cancer group showed significantly higher MIF level than the non-cancer counterpart (9.37±1.57 vs. 3.66±0.49, mean ± standard error, *P* = 0.001). Tissue MIF level varied remarkably between *H*. *pylori*-positive and -negative groups: the MIF level in the *H*. *pylori*-positive group was significantly elevated in the cancer subgroup than in control (*P* = 0.012) or dysplasia (*P* < 0.01) subgroups. (Fig 1A, S1 Table see online). *H*. *pylori*-positive cancer subgroup also showed significantly higher MIF level than *H*. *pylori*-negative control (*P* < 0.01) (Fig 1A, S1 Table). In contrast, in *H*. *pylori*-negative group, there was no significant difference in MIF level between control, dysplasia and cancer subgroups (*P* > 0.05). (Fig 1A, S1 Table)

### Tissue LC3A and LC3B levels

**Fig 2:**
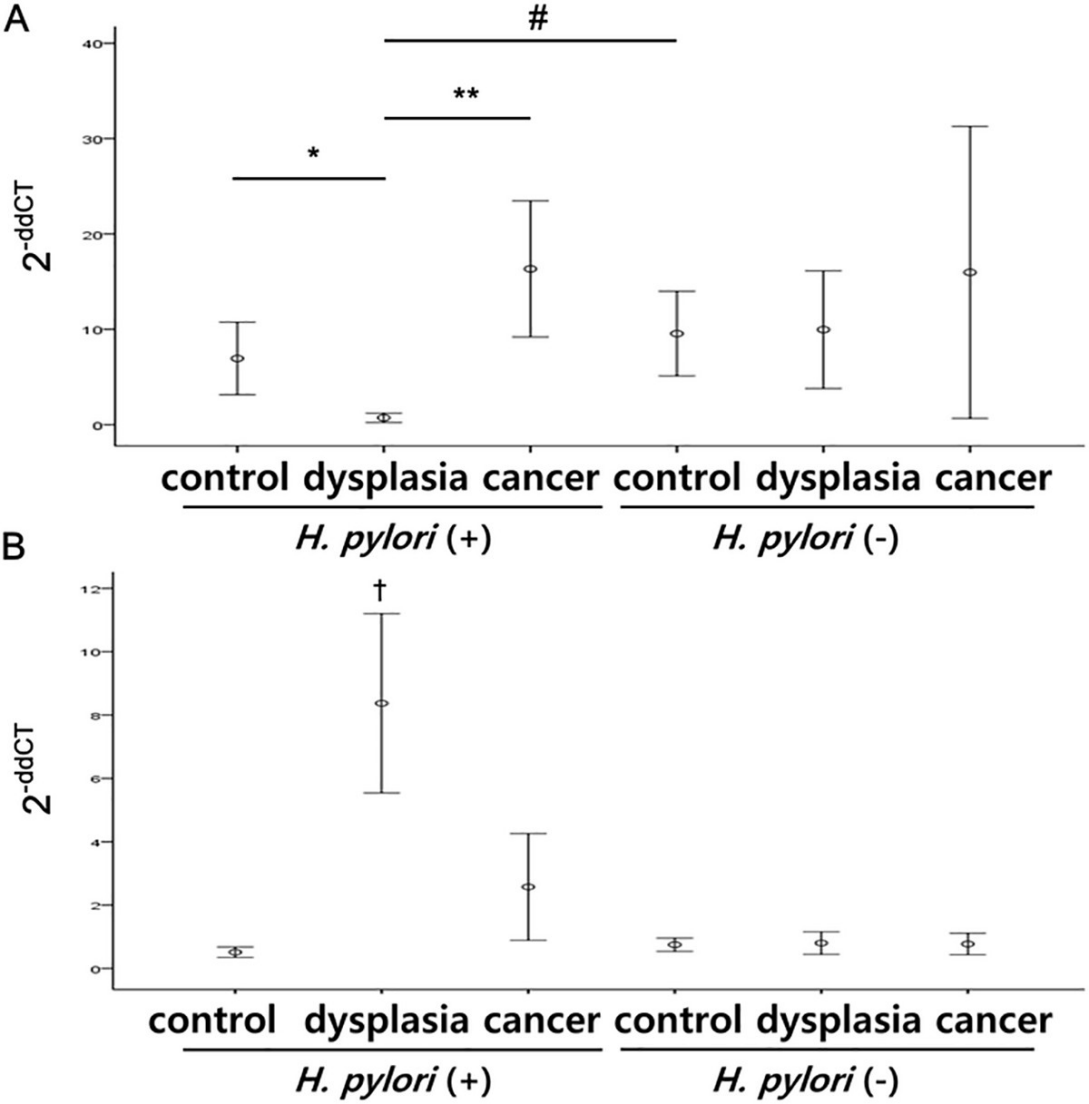
Tissue LC3A and LC3B levels. (A) *H*. *pylori*-positive dysplasia subgroup showed significantly lower level of LC3A level than that of *H*. *pylori-*positive control, cancer, and *H*. *pylori*-negative control subgroups. (B) *H*. *pylori*-positive dysplasia subgroup showed significantly higher level of LC3B than every other subgroup. *P < 0.05, **,^#^*P* < 0.01, ^†^*P* < 0.001, except to *H*. *pylori*-positive cancer group, *P* = 0.01.

Similar to MIF, LC3A level also showed no significant difference between *H*. *pylori-*negative subgroups of control, dysplasia and cancer (*P* > 0.05) (Fig 2A, S1 Table see online). However, *H*. *pylori*-positive dysplasia subgroup showed significantly lower levels of LC3A level than *H*. *pylori-*positive control (*P* = 0.025), cancer (*P* < 0.01) and *H*. *pylori*-negative control (*P* < 0.01) subgroups (Fig 2A, S1 Table). *H*. *pylori*-positive dysplasia subgroup showed significantly higher levels of LC3B than other subgroups including *H*. *pylori*-negative ones. (*P* < 0.05) (Fig 2B, S1 Table see online).

### Tissue Atg5 level

Within the *H*. *pylori*-positive group, a statistically significant trend was observed in the levels of Atg5 increasing sequentially from control to dysplasia, and to cancer subgroups (each *P* < 0.05) (Fig 1B, S1 Table). In contrast, no significant difference in Atg5 level was seen within *H*. *pylori-*negative group (*P* > 0.05) (Fig 1B, S1 Table see online).

*H*. *pylori*-negative control subgroup showed significantly higher Atg5 level than *H-pylori*-positive control subgroup. (*P* < 0.05) (Fig 1B, S1 Table). *H*. *pylori-*negative dysplasia subgroup also showed significantly higher expression compared with *H-pylori*-positive dysplasia subgroup. (*P* < 0.05) (Fig 1B, S1 Table see online)

### MIF and autophagy markers

Multiple linear regression showed that the autophagy markers (LC3A, LC3B, and Atg5) predicted MIF level with adjusted R^2^ = 0.492 (*P* < 0.001) (Table 2). No multi-collinearity between the variables was seen (VIF < 10, VIF: variance inflation factor).

**Table 2:** Multiple linear regression.

		B	ß	t	*P*	VIF
**MIF**	**LC3A**	0.227	0.469	12.105	**< 0.01**	1.335
	**LC3B**	0.725	0.346	10.292	**< 0.01**	1.007
	**Atg5**	0.264	0.224	5.786	**< 0.01**	1.339

LC3A, LC3B, and Atg5 predicted MIF level with adjusted R2 = 0.492, P < 0.001; B: unstandardized coefficients; ß: standardized coefficients; VIF: variance inflation factor

### Longitudinal changes in tissue MIF expression

**Fig 3:**
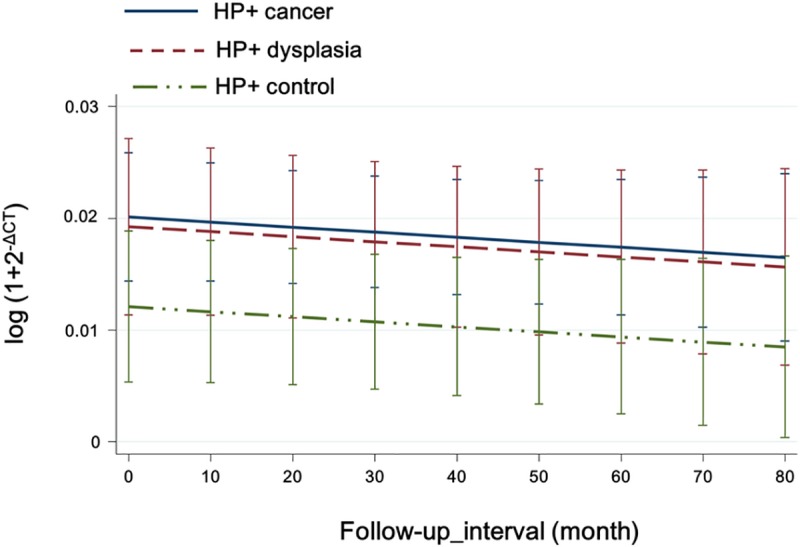
Longitudinal changes in MIF level. *H*. *pylori*-eradicated cancer group showed significantly higher MIF level than *H*. *pylori*-eradicated control group (*P* < 0.05), which remained constant throughout the follow-up period. No significant change in the MIF level over time was seen. (*P*>0.05).

Among the enrolled patients, 386 patients were followed-up at least once with MIF PCR of gastric tissue. The mean follow-up period was 45.52 months, and the mean interval between endoscopic biopsies was 15.88 months. For statistical analysis, 38 patients who tested *H*. *pylori*-positive and failed to eradicate the pathogen were excluded. (S2 Table see on line) Among 280 *H*. *pylori*-positive patients and successfully eradicated, 198 (56.9% of total) had IM. In both *H*. *pylori*-positive and -negative groups, there was no significant temporal change in tissue MIF level (*P* > 0.05) (Fig 3, S1 Fig see online). *H*. *pylori*-positive cancer group showed significantly higher MIF levels than *H*. *pylori*-positive control group, which remained constant throughout the follow-up period (*P* < 0.05) (Fig 3).

No statistically significant longitudinal difference in MIF levels was seen between control, dysplasia and cancer subgroups within both *H*. *pylori* -positive and -negative groups (*P* > 0.05) (S1 Fig see online). According to IM status, there was no significant longitudinal change in MIF expression (*P* > 0.05)

### Longitudinal changes in Atg5 expression

**Fig 4:**
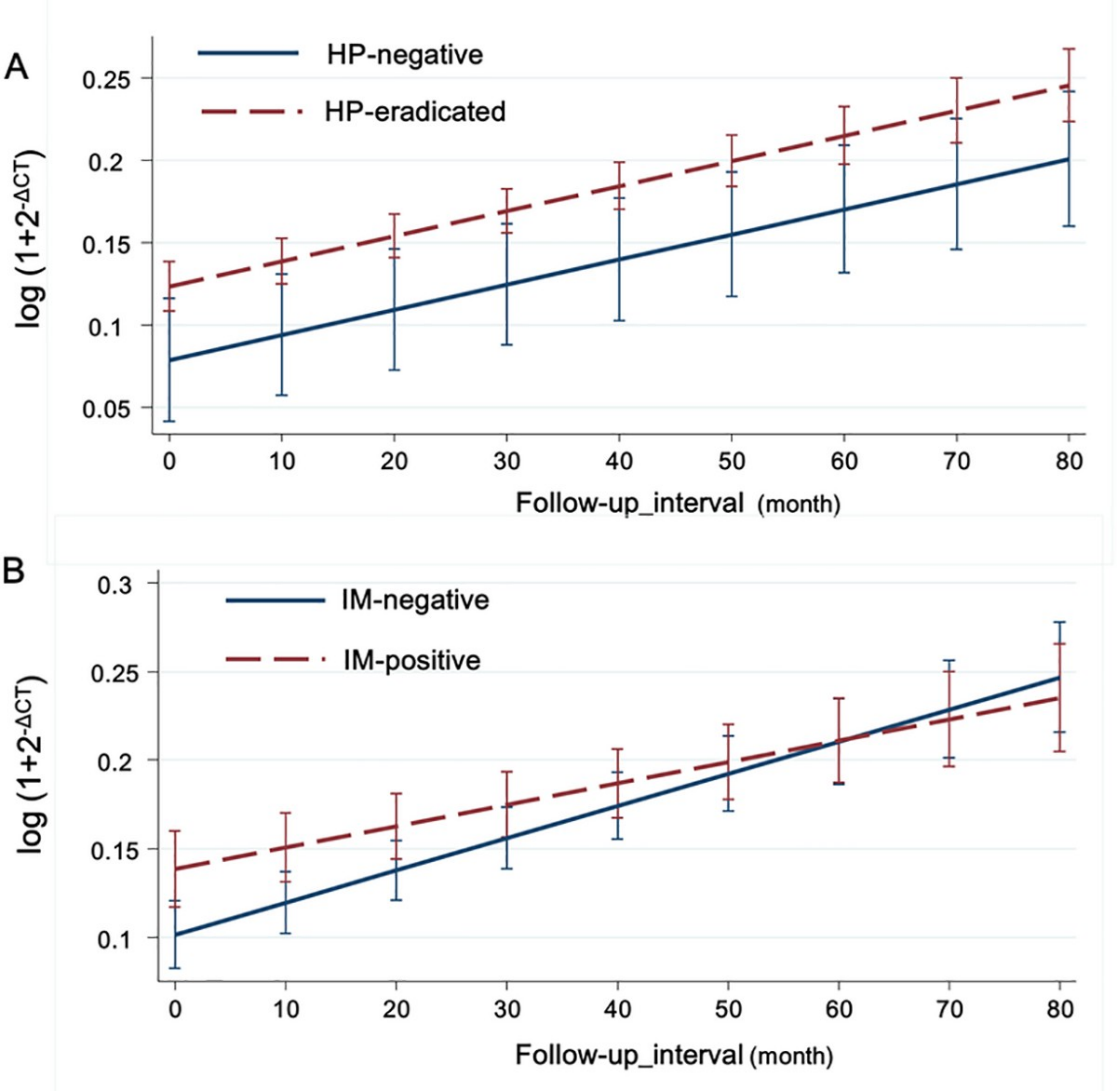
Longitudinal changes in Atg5 level according to *H*. *pylori* status and intestinal metaplasia (IM) status. (A) The expression of Atg5 longitudinally increased in both *H*. *pylori*-eradicated and -negative groups (*P* < 0.05). Atg5 expression remained constantly higher over time in *H*. *pylori*-eradicated group than in *H*. *pylori*-negative group (*P* = 0.017). (B) IM-positive group showed initially higher expression of Atg5 than IM-negative group (*P* < 0.05). However, the rate of increase was significantly lower than in IM-negative group, and the reversal of the expression level was noted eventually (*P* < 0.05).

Among the enrolled patients, we serially obtained gastric tissue for Atg5 PCR from 319 patients. The mean follow-up period was 38.24 months and the mean interval between endoscopic biopsies was 18.39 months. For statistical analysis, 17 patients who were *H*. *pylori*-positive and failed to eradicate it were excluded. One hundred and twenty-one (40.1% of total) out of 262 *H*. *pylori*-positive patients showed intestinal metaplasia (IM) histologically (S2 Table). The expression of Atg5 gradually increased in both *H*. *pylori*-eradicated and -negative groups (*P* < 0.05) (Fig 4A). Atg5 expression remained constantly higher with time in *H*. *pylori*-eradicated group than in *H*. *pylori*-negative group (*P* = 0.017) (Fig 4A). IM-positive group showed initially higher expression of Atg5 than IM-negative group. Atg5 expression increased gradually in both groups. However, as the rate of increase was significantly lower in the IM-positive group, the expression levels were reversed eventually (Fig 4B). The differential rate of increase was statistically significant (*P* < 0.05) (Fig 4B). No statistically significant longitudinal difference was seen in Atg5 levels between control, dysplasia and cancer subgroups within the *H*. *pylori*-positive and -negative groups (*P* > 0.05) (S5 Table see online)

## Discussion

MIF level was significantly elevated in cancer subgroup than in control or dysplasia subgroups of patients, only within *H*. *pylori*-positive group. Within the *H*. *pylori*-positive group, the LC3A and LC3B levels showed significant differences within *H*. *pylori*-positive subgroups. Similarly, Atg5 expression increased sequentially from control to dysplasia, and to cancer subgroups within the *H*. *pylori*-positive group. Multiple linear regression analysis showed autophagy markers (LC3A, LC3B, and Atg5) directly predicted MIF level (adjusted R^2^ = 0.492, *P* < 0.001). Serial follow-up showed longitudinal increase in Atg5 level in general, with constantly higher levels in *H*. *pylori*-eradicated group than in -negative group. Taken together, our results suggest the role of MIF and autophagy markers and their interaction in *H*. *pylori*-associated gastric carcinogenesis. Indeed, this is the first report suggesting an important distinction between *H*. *pylori*-positive and -negative gastric tumorigenesis in terms of MIF and autophagy.

As described in detail previously with regard to *H*. *pylori* and MIF [6], tumor microenvironment is an important concept in tumorigenesis. Among the factors known to be involved, MIF is related to various types of malignancy [7, 24]. MIF plays a role in angiogenesis, lymph node metastasis and distant metastasis [4, 25]. In addition, MIF is involved in signal transduction and stimulates ERK1, and ERK2 MAP kinase, which are related to carcinogenesis [26]. Furthermore, MIF mediates cell proliferation, especially through Ras-related signaling pathway. Meanwhile, MIF negatively affects tumor suppressor p53 by inhibiting its anti-proliferative property. High concentrations of MIF expressed by dysplastic or inflammatory cells bypass the p53 pathway, accumulate mutations via cellular proliferation, prolong cellular life span, and inhibit cell death [4, 27–30]. MIF also inhibits the activity of p21, cyclin G1, and Mdm2 [26]. Furthermore, the angiogenic activity of MIF is established from the interaction between MIF and CXC chemokines, Interleukin (IL)-8 or VEGF [29, 31]. The relationship between *H*. *pylori* and MIF has been investigated in vitro, and clinically with human tissue samples. In gastric mucosa, the increased expression of MIF by epithelial cells, T cells, and macrophages was reported to be associated with *H*. *pylori* infection. The difference in distribution of MIF-positive cells between antrum and corpus was also reported [32, 33]. In vitro cell culture studies showed that *H*. *pylori* directly stimulated MIF secretion from monocytes via *cag* PAI expression, resulting in gastric cell proliferation [33]. The effect was blocked with anti-MIF antibody, suggesting the role of MIF as a mediator of *H*. *pylori*-induced tumorigenesis [34–37]. The progressive increase of epithelial and serum MIF levels in *H*. *pylori*, was associated with gastritis, intestinal metaplasia, and gastric cancer, respectively. It suggested the potential role of MIF as a biomarker of gastric cancer [38]. Significant relationship between *H*. *pylori* and MIF is further supported by the reduced levels of MIF following eradication of *H*. *pylori* [35]. Another clinical study showed that serum MIF levels in patients had better diagnostic value than carcinoembryonic antigen (CEA) and even correlated with the 5-year survival when combined with CEA [39]. MIF was also up-regulated in a rat model of acute gastric ulcer [40]. *H*. *pylori* infection releases MIF-induced phosphorylation of epidermal growth factor receptor (EGFR) [32]. However, another report suggested that MIF expression and secretion did not directly increase after *H*. *pylori* infection, although IL-8 expression and secretion were upregulated [37].

In the current study, no significant difference in the tissue MIF level was found based on *H*. *pylori* status alone. However, significant differences were found considering both pathology and infection status. In *H*. *pylori*-positive group, the MIF level was significantly elevated in cancer subgroup than in control or dysplasia subgroups. In contrast, in *H*. *pylori*-negative group, there was no significant difference in MIF level between control, dysplasia and cancer subgroups. These findings imply that MIF regulated the critical transformation from dysplasia to cancer only in *H*. *pylori*-positive gastric tissue. Based on in vitro studies reported previously [33], *H*. *pylori* stimulated MIF secretion through its *cag* PAI, especially in the tumor microenvironment from dysplasia to cancer. Lack of variation in MIF levels in *H*. *pylori*-negative group according to its pathological transformation suggested that MIF partly explains the distinct pathogenesis of *H*. *pylori*-positive cancer compared with -negative neoplasm. We also investigated longitudinal changes in tissue MIF level upon follow-up considering *H*. *pylori* status and pathology. Few clinical studies monitored tissue MIF levels over a period of time. In both *H*. *pylori*-positive and -negative groups, there was no significant temporal variation in the MIF level. The *H*. *pylori*-positive cancer group showed significantly higher levels of MIF than control and dysplasia subgroups of patients, throughout the follow-up period. Changes in IM status were also analyzed. However, we found no significant longitudinal differences between the groups. All the patients with *H*. *pylori* infection underwent eradication therapy, and therefore, these findings imply that the baseline MIF level was stable once established.

Autophagy is a cellular degradation process that maintains intracellular homeostasis via lysosomal degradation of cytoplasmic constituents and recycling of amino acids and energy [41]. Autophagy plays a dual role as a tumor suppressor and a protector of cancer cell survival [41]. MIF and autophagy have been linked by several studies. MIF was shown to play a permissive role in the maintenance of cardiac contractile function under starvation by regulation of autophagy [42]. In breast cancer research, regulation of MIF expression and suppression of autophagic cell death is a potent mechanism contributing to chemoresistance and tumorigenicity [43]. Low expression of Beclin-1, a well-known marker of autophagy, associated with high Bcl-xL was shown to predict a malignant phenotype and poor prognosis of stomach cancer [14]. In contrast, high expression of another autophagy marker LC3 was observed in gastrointestinal cancers including gastric cancer. Interestingly, LC3 immunoreactive score gradually increased during early carcinogenesis, while it remained constant in later progression [13]. Isoforms of LC3 (LC3A, B and C) are structural proteins of autophagosomal membranes. Whether each LC3 protein has a similar biological role in autophagy remains obscure. LC3A showed a perinuclear and nuclear localization, while LC3B was equally distributed throughout the cytoplasm and localized in the nucleolar regions [44]. In oral squamous cell carcinoma, increased LC3B expression was associated with aggressive clinicopathological features and unfavorable prognosis [45]. In our study, LC3A and LC3B levels varied significantly in subgroups according to *H*. *pylori* status. In *H*. *pylori*-positive group, the LC3A level was significantly lower in the dysplasia subgroup than in control or cancer subgroup. In contrast, the LC3B level showed higher levels in the dysplasia subgroup than in control or cancer. No significant difference in either LC3A or LC3B was observed within the *H*. *pylori*-negative group. This result is interesting because only *H*. *pylori*-positive dysplasia group showed significant difference in LC3A and LC3B levels. *H*. *pylori* infection might play a role in the progression from control to dysplasia and/or dysplasia to cancer via autophagy, with subtle difference in the location of effect within the cell structure represented by the markers LC3A and LC3B. Our novel finding regarding the isoforms of LC3 may elucidate the complex relationship between autophagy and cancer, with a possible role in dysplasia.

Atg5 is another autophagy marker involved in the early stages of autophagosome [46]. In studies with melanoma and non-small cell lung cancer, Atg5 was shown to play an antitumor role especially in early carcinogenesis [47, 48] In contrast, in pancreatic cancer, autophagy is actually required for tumorigenesis de novo. Genetic inactivation of Atg5 was used to demonstrate their theory [49]. We observed that the tissue levels of Atg5 increased gradually from control, dysplasia and cancer in *H*. *pylori*-positive group. Within the *H*. *pylori-*negative group, no significant difference in the level was seen. Our result suggests that increased Atg5 activity may play a role in gastric carcinogenesis, in *H*. *pylori-*infected patients. Mutational or expressional alteration of Atg5 gene in gastrointestinal cancers was reported previously [50], suggesting that Atg5 expression in our study resulted in similar outcomes. This finding further implies that the distinct features of *H*. *pylori-*positive dysplasia and cancer could be attributed partly to MIF and autophagy.

In the longitudinal analysis of Atg5, we found a gradual increase in Atg5 expression in both *H*. *pylori*-positive and -negative groups. The level of Atg5 expression remained constantly higher in *H*. *pylori*-positive group than in *H*. *pylori*-negative group. The role of autophagy in aging was reported previously [51]. Modulation of key autophagic components such as Ulk3, Atg5, or Atg7 has been shown to control senescence, possibly through Pi3K-Akt-mTOR pathway, limiting oncogene signaling and enabling cell cycle exit [52]. However, studies with a serial follow-up of the markers in human gastric tissue are scarce. The current result directly showing the increased expression of Atg5 over time explains the role of autophagy in human aging.

In terms of IM, the IM-positive group showed initially higher Atg5 level than the IM-negative group. Atg5 level increased over time in both groups. However, the rate of increase was lower in the IM-positive group than in IM-negative group. Interestingly, it resulted in the cross of the line of expression eventually. The long-term effect of *H*. *pylori* eradication on autophagy regarding IM may be inferred from our findings, which is a unique implication of our study.

Based on similarity in distribution of tissue levels of MIF and autophagy markers within the *H*. *pylori*-positive group, we directly correlated MIF and autophagy markers. Multiple linear regression analysis showed that the autophagy markers (LC3A, LC3B, and Atg5) predicted MIF level with relatively high adjusted R-square value providing indirect evidence for the relationship between MIF and autophagy in human gastric pathology.

In conclusion, we found that the tissue expression of MIF and autophagy markers LC3A, LC3B and Atg5 showed significant differences within *H*. *pylori*-positive subgroups, but not within the *H*. *pylori*-negative counterpart. The *H*. *pylori*-positive dysplasia subgroup showed a distinct pattern of tissue levels compared with other subgroups regarding LC3A and LC3B. Atg5 expression gradually increased over time. After *H*. *pylori* eradication, the Atg5 levels in the IM group were lower than in IM-negative counterpart. A direct baseline correlation between MIF and the autophagy markers was observed in human gastric tissue, suggesting a role in gastric carcinogenesis in *H*. *pylori*-infected gastric tissue.

## Supporting information

S1 Fig Longitudinal changes in MIF level of *H*. *pylori*-negative subgroups. No statistically significant longitudinal difference was noted. (P > 0.05).
(TIF)

S1 Table Expression of MIF and autophagy markers. (DOCX)

S2 Table Number of followed-up patients. (DOCX)

S3 Table Expression level of MIF and autophagy markers. (DOCX)

S4 Table Primer sequences for qRT- PCR. (DOCX)

S5 Table **(A)** Linear mixed model result of Atg5 levels for *H*. *pylori*-eradicated subgroups. Base reference was *H*. *pylori*-eradicated control subgroup. Longitudinal change according to follow-up months of *H*. *pylori*-eradicated dysplasia and cancer group was compared with the reference subgroup. (B)Linear mixed model result of Atg5 levels for *H*. *pylori*-negative subgroups. Base reference was *H*. *pylori*-negative control subgroup. Longitudinal change according to follow-up months of *H*. *pylori*-negative dysplasia and cancer group was compared with the reference subgroup.
(DOCX)

## Abbreviations

MIF: macrophage migration inhibitory factor; *H*. *pylori*, *Helicobacter pylori*
Atg5: autophagy related gene 5
LC3A and LC3B: microtubule-associated protein light chain 3
EGC: early gastric cancer; AGC, advanced gastric cancer; IM, intestinal metaplasia
