# Supplementary figures and images for: Correlation between macrophage migration inhibitory factor and autophagy in Helicobacter pylori-associated gastric carcinogenesis
Source: PLoS One. 2019 Feb 11;14(2):e0211736. doi: 10.1371/journal.pone.0211736 (PMC6370197; doi:10.1371/journal.pone.0211736)

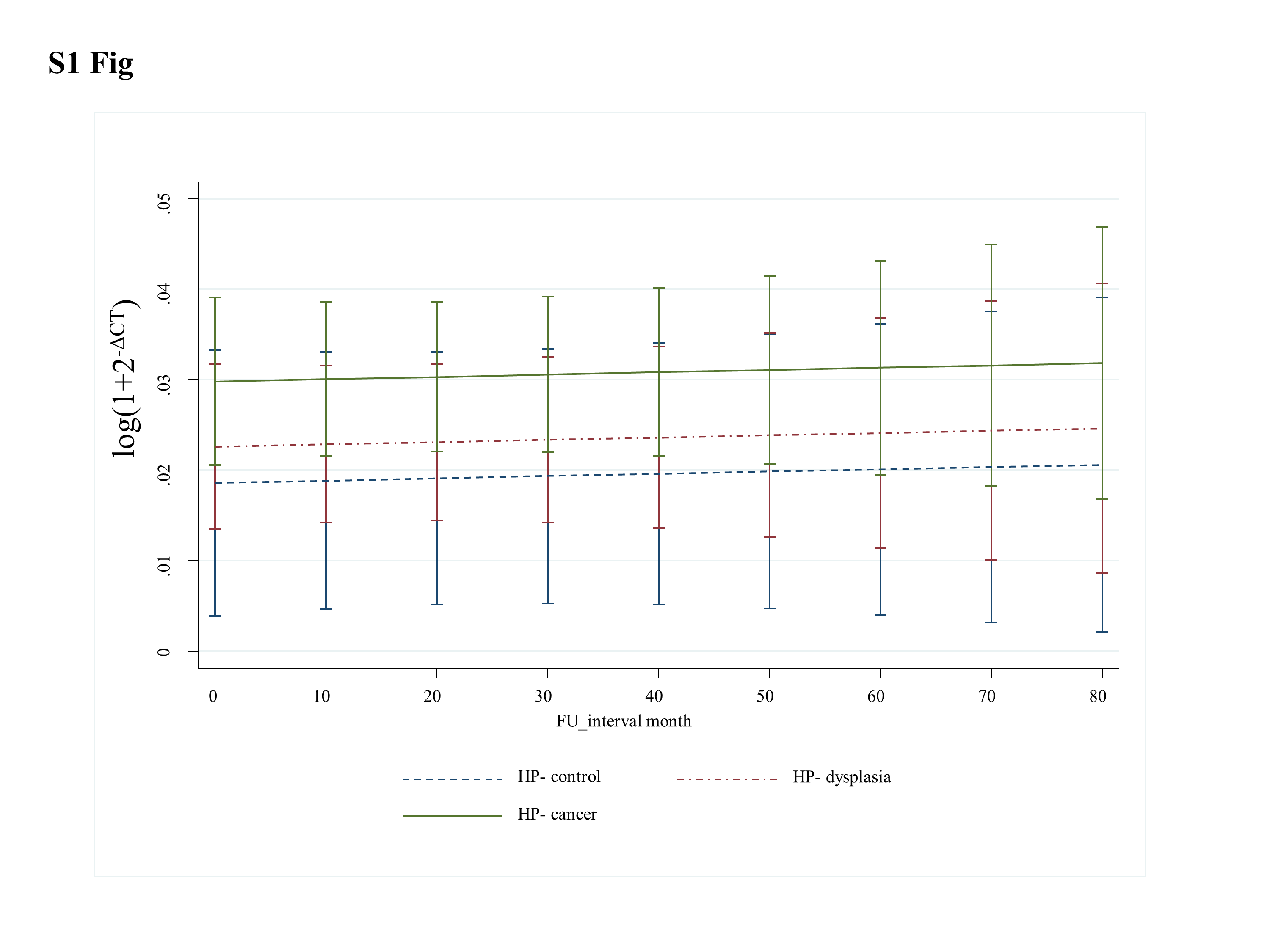

Supplement: S1 Fig — (P > 0.05). (TIF) [file pone.0211736.s001.Tif]
