# Supplementary material for: Correlation between macrophage migration inhibitory factor and autophagy in Helicobacter pylori-associated gastric carcinogenesis
Source: PLoS One. 2019 Feb 11;14(2):e0211736. doi: 10.1371/journal.pone.0211736 (PMC6370197; doi:10.1371/journal.pone.0211736)
Supplement: S1 Table — (DOCX) [file pone.0211736.s002.docx]

**S1 Table** Expression of MIF and autophagy markers

|  |  | ***N*** |  | **2^ΔΔCT** |  |  |
| --- | --- | --- | --- | --- | --- | --- |
|  |  |  | MIF | LC3A | LC3B | Atg5 |
|  | control | 84 | 3.5 ± 0.72 | 6.94 ± 1.90 | 0.51 ± 0.83 | 0.94 ± 0.10 |
| **HP positive** | dysplasia | 49 | 2.02 ± 0.52 | **0.72 ± 0.24*** | **8.37 ± 1.41*** | 2.31 ± 0.42 |
|  | cancer | 170 | **10.27 ± 1.86*** | 16.33 ± 3.57 | 2.57 ± 0.84 | 7.91 ± 1.33 |
| *HP positive total* | | *303 (66.9%)* | 7.1 ± 1.09 | 11.20 ± 2.10 | 2.94 ± 0.54** | 5.07 ± 0.77† |
|  | control | 81 | 2.98 ± 0.58 | 9.54 ± 2.22 | 0.75 ± 0.11 | 6.55 ± 1.37 |
| **HP negative** | dysplasia | 33 | 6.4 ± 2.25 | 9.96 ± 3.08 | 0.80 ± 1.78 | 10.61 ± 2.42 |
|  | cancer | 36 | 6.53 ± 2.02 | 15.96 ± 7.65 | 0.77 ± 0.17 | 10.07 ± 3.16 |
| *HP negative total* | | *150 (33.1%)* | 4.59 ± 0.77 | 11.18 ± 2.29 | 0.77 ± 0.08** | 8.29 ± 1.19† |
| *Total* |  | 453 (100%) | 6.24 ± 0.77 | 11.19 ± 1.59 | 2.22 ± 0.37 | 6.14 ± 0.65 |

HP, *Helicobacter pylori*.

*Bold style indicates remarkable statistical significance compared with other groups; Data shown in Mean ± Standard error .For MIF, *H. pylori*-positive cancer subgroup showed significantly higher expression than *H. pylori* positive control (*P* = 0.012), *H. pylori* positive dysplasia (*P* < 0.01),and H. pylori negative control subgroup (*P* = 0.003). For LC3A, *H. pylori*-positive dysplasia subgroup showed significantly lower level than *H. pylori-*positive control (*P* = 0.025), cancer (*P* < 0.01) and *H. pylori*-negative control (*P* < 0.01) subgroups. For LC3B, *H. pylori*-positive dysplasia subgroup showed significantly higher level than all other subgroups including H. pylori-negative ones.

For Atg5, the significant increase in the expression from *H. pylori*-positive control to *H. pylori* dysplasia (*P* = 0.028) and from dysplasia to *H. pylori*-positive cancer subgroup (*P* = 0.001). *H. pylori*-positive control also showed significantly different level of expression from *H. pylori*-negative control and *H. pylori*-negative.

^**, †^ :*P* < 0.05, between *H. pylori*-positive and -negative groups
