# Supplementary material for: Correlation between macrophage migration inhibitory factor and autophagy in Helicobacter pylori-associated gastric carcinogenesis
Source: PLoS One. 2019 Feb 11;14(2):e0211736. doi: 10.1371/journal.pone.0211736 (PMC6370197; doi:10.1371/journal.pone.0211736)
Supplement: S2 Table — (DOCX) [file pone.0211736.s003.docx]

**S2 Table** Number of followed-up patients

|  |  | MIF | | Atg5 | |
| --- | --- | --- | --- | --- | --- |
|  |  | N | % | N | % |
|  | Control | 127 | 36.5 | 85 | 28.1 |
| HP+ | Dysplasia | 54 | 15.5 | 53 | 17.5 |
|  | Cancer | 99 | 28.4 | 124 | 41.1 |
|  | IM*(%) | 198 | 56.9 | 121 | 40.1 |
| *HP+ total* |  | 280^**^ | 80.6 | 262^**^ | 86.8 |
|  | Control | 11 | 3.2 | 24 | 7.9 |
| HP- | Dysplasia | 23 | 6.6 | 8 | 2.6 |
|  | Cancer | 34 | 9.8 | 8 | 2.6 |
| *HP- total* |  | 68 | 19.5 | 40 | 13.2 |
| Total |  | 348 | 100.0 | 302 | 100.0 |

^*^IM: intestinal metaplasia;

^**^Only patients with successful eradication are included
