# Supplementary material for: Correlation between macrophage migration inhibitory factor and autophagy in Helicobacter pylori-associated gastric carcinogenesis
Source: PLoS One. 2019 Feb 11;14(2):e0211736. doi: 10.1371/journal.pone.0211736 (PMC6370197; doi:10.1371/journal.pone.0211736)
Supplement: S3 Table — (DOCX) [file pone.0211736.s004.docx]

**S3 table** Expression level of MIF and autophagy markers

| Sex | N | % | MIF | LC3A | LC3B | Atg5 |
| --- | --- | --- | --- | --- | --- | --- |
| Male | 273 | (60.3) | 6.26±0.91 | 12.68±2.39 | 2.16±0.33 | 6.02±0.82 |
| Female | 180 | (39.7) | 6.19±1.36 | 8.92±1.67 | 2.30±0.77 | 6.31±1.07 |
| P value |  |  | 0.963 | 0.200 | 0.860 | 0.832 |

Values are shown as Mean ± Standard error
