# Supplementary material for: Correlation between macrophage migration inhibitory factor and autophagy in Helicobacter pylori-associated gastric carcinogenesis
Source: PLoS One. 2019 Feb 11;14(2):e0211736. doi: 10.1371/journal.pone.0211736 (PMC6370197; doi:10.1371/journal.pone.0211736)
Supplement: S4 Table — (DOCX) [file pone.0211736.s005.docx]

**S4 Table** Primer sequences for qRT- PCR

| Gene name | Forward primer | Reverse primer | Size  (bp) |
| --- | --- | --- | --- |
| *MIF* | 5-AGA ACC GCT CCT ACA GCA AG-3 | 5-TAG GCG AAG GTG GAG TTG TT-3 | 133 |
| *LC3A* | 5-CCA GCA AAA TCC CGG TGA-3 | 5-TGG TCC GGG ACC AAA AAC T-3 | 88 |
| *LC3B* | 5-ACC ATG CCG TCG GAG AAG-3 | 5-GGT TGG ATG CTG CTC TCG AA-3 | 90 |
| *Atg5* | 5-ATG CAG GGA ACA CTA AGC TG-3 | 5-TCT AGG GCA TTG TAG GCT TG-3 | 104 |
| *ACTB* (β-actin) | 5-TTC GAG CAA GAG ATG GCC AC-3 | 5-CGG ATG TCC ACG TCA CAC TT-3 | 202 |

qRT- PCR, quantitative real time-polymerase chain reaction; bp, base pair.
