# Supplementary material for: Correlation between macrophage migration inhibitory factor and autophagy in Helicobacter pylori-associated gastric carcinogenesis
Source: PLoS One. 2019 Feb 11;14(2):e0211736. doi: 10.1371/journal.pone.0211736 (PMC6370197; doi:10.1371/journal.pone.0211736)
Supplement: S5 Table — (A) Linear mixed model result of Atg5 levels for H. pylori-eradicated subgroups. Base reference was H. pylori-eradicated control subgroup. Longitudinal change according to follow-up months of H. pylori-eradicated dysplasia and cancer group was compared with the reference subgroup. (B)Linear mixed model result of Atg5 levels for H. pylori-negative subgroups. Base reference was H. pylori-negative control subgroup. Longitudinal change according to follow-up months of H. pylori-negative dysplasia and cancer group was compared with the reference subgroup. (DOCX) [file pone.0211736.s006.docx]

**S5 table (A)** Linear mixed model result of Atg5 levels for H. pylori-eradicated subgroups. Base reference was H. pylori-eradicated control subgroup. Longitudinal change according to follow-up months of H. pylori-eradicated dysplasia and cancer group was compared with the reference subgroup. **(B)** Linear mixed model result of Atg5 levels for *H. pylori*-negative subgroups. Base reference was *H. pylori*-negative control subgroup. Longitudinal change according to follow-up months of *H. pylori*-negative dysplasia and cancer group was compared with the reference subgroup.

**(A)**

|  | Coef | S.E. | z | P>\|z\| | 95% C.I | |
| --- | --- | --- | --- | --- | --- | --- |
| Dysplasia | 0.02 | 0.019 | 1.19 | 0.236 | -0.015 | 0.059 |
| cancer | -0.025 | 0.016 | -1.57 | 0.116 | -0.058 | 0.006 |

Coef, coefficient; S.E., standard error, C.I., confidence interval

**(B)**

|  | Coef | S.E. | z | P>\|z\| | 95% C.I | |
| --- | --- | --- | --- | --- | --- | --- |
| Dysplasia | 0.06 | 0.074 | 0.83 | 0.406 | -0.08 | 0.21 |
| cancer | 0.046 | 0.075 | 0.63 | 0.531 | -0.09 | 0.19 |

Coef, coefficient; S.E., standard error, C.I., confidence interval
